# Evaluation of C-reactive protein as predictor of adverse prognosis in acute myocardial infarction after percutaneous coronary intervention: A systematic review and meta-analysis from 18,715 individuals

**DOI:** 10.3389/fcvm.2022.1013501

**Published:** 2022-11-16

**Authors:** Shijie Liu, Hongcheng Jiang, Menaka Dhuromsingh, Lei Dai, Yue Jiang, Hesong Zeng

**Affiliations:** ^1^Division of Cardiology, Department of Internal Medicine, Tongji Hospital, Tongji Medical College, Huazhong University of Science and Technology, Wuhan, Hubei, China; ^2^Hubei Provincial Engineering Research Center of Vascular Interventional Therapy, Wuhan, Hubei, China

**Keywords:** acute myocardial infarction, percutaneous coronary intervention (PCI), C-reactive protein (CRP), adverse prognosis, inflammatory/anti-inflammatory factors, coronary arterial disease

## Abstract

**Background:**

Proper prognostic biomarker is of great importance for clinical decision-making in patients with acute myocardial infarction (AMI) undergoing percutaneous coronary intervention (PCI). Although recently emerges plenty of novel inflammatory biomarkers, the canonical inflammatory mediator C-reactive protein still plays an important role in prognosing adverse post-infarction complications.

**Methods:**

PubMed, Embase, and Medline were systematically searched from the establishment of databases up to December 2021, conforming with standards set forth by the Preferred Reporting Items for Systematic Reviews and Meta-Analyses (PRISMA) statement.

**Results:**

A total of 23 studies were eventually eligible for this meta-analysis, including 18,715 individuals. Our findings showed that elevated C-reactive protein (CRP) had a statistically significant superiority in predicting all-cause mortality (OR: 3.22, 95% CI: [2.71, 3.84], *p* < 0.00001), cardiovascular death (OR: 3.26, 95% CI: [2.30, 4.61], *p* < 0.00001), major adverse cardiovascular events (MACEs) (OR: 2.85, 95% CI [2.08, 3.90], *p* < 0.00001), heart failure (OR: 2.29, 95% CI: [1.48, 3.54], *p* = 0.0002), recurrent myocardial infarction (OR: 1.76, 95% CI: [1.28, 2.43], *p* < 0.001), and restenosis (OR: 1.71, 95% CI: [1.18, 2.47], *p* = 0.004). Subgroup analysis implies that CRP had better performance in predicting plenty of hospitalization and short-term (<12 months) adverse prognosis than long-term prognosis and Asian patients with elevated CRP were under more risk in adverse prognosis after PCI than Europeans.

**Conclusion:**

Our meta-analysis suggests that CRP is a prospective predictor of the prognosis in patients with AMI undergoing PCI, especially in hospitalization and short-term and in the Asian group.

## Introduction

Despite advances in the therapies for coronary artery disease, such as percutaneous coronary intervention (PCI) which has saved a lot of lives since its application (1), myocardial infarction is still the main cause of morbidity and mortality among all cardiovascular diseases worldwide (2, 3). For patients with acute myocardial infarction (AMI) undergoing PCI, inflammatory response plays a pivotal role in the whole pathophysiological process. Since inflammation can promote endothelial cell injury (4), vascular remodeling (5), and plaque destabilization (6), it is now regarded as another risk factor for AMI besides traditionally acknowledged risk factors like hypertension and dyslipidemia (7). In addition, the pro-inflammatory response occurs in the early stage of AMI and may exacerbate after PCI, thus contributing to the death of myocardial cells (8).

During AMI, the release of the intracellular content and destruction of the extracellular matrix (9) leads to the generation of a cascade of inflammatory infiltration and mediators (10). Biomarkers from inflammatory infiltration are based on the accumulation of plasma inflammatory cells (11) [e.g., neutrophil-to-lymphocyte ratio (NLR) (12), systemic immune-inflammation index (SII) (13), and novel subclasses of Tregs (14)], which are easily influenced by heterogeneity under ethnicity and individuals. With respect to inflammatory mediators, plasma molecules consisting of common inflammatory pathways are routinely measured in clinical practice and have been studied as prognostic biomarkers, such as IL-1b (15) or cytokine concentrations and proinflammatory to anti-inflammatory cytokine ratios (16). However, some limitations like a small sample size are unneglectable in all these studies, requiring for in-depth investigation. It is noteworthy that C-reactive protein (CRP), among plenty of canonical inflammatory mediators, has exhibited excellent capacity in predicting post-STEMI adverse prognoses in comprehensive comparison studies (16–19). It might result from that CRP, as a canonical downstream of multiple inflammatory pathways (8, 17, 18, 20), actively participating in the inflammatory process (21), stimulating the secretion of various pro-inflammatory cytokines, promoting the release of reactive oxygen, and inducing the switch of quiescent macrophage to pro-inflammatory M1 subtype (22). Even the relationship between the prognosis of AMI and other novel biomarkers has been extensively explored, such as plasma long pentraxin-3 (23) and YKL-40 (24), but none have been proven as practically useful as hsCRP.

Hence, CRP is a typically and frequently used biomarker of inflammation, which may give rise to an increased cardiovascular risk (6). The relationship between the increase of plasma CRP concentration and the prognosis of patients with AMI, such as mortality (25, 26), cardiovascular mortality (27, 28), the rate of major adverse cardiovascular events (MACEs) (29, 30), heart failure (5), recurrent myocardial infarction (31, 32), and restenosis (33, 34), has been thoroughly studied in the past decades. A single study alone can only partially depict the whole profile of the association between CRP and poor prognosis in AMI after CRP. Besides, although Mincu et al. (35) had made a persuasive meta-analysis in 2016 including seven studies and 6,993 patients, the detailed subgroup analyses remain to be in-depth investigated. Therefore, we conducted this meta-analysis in an attempt to elucidate the relationship between the elevation of CRP and the prognosis of patients with AMI undergoing PCI and figure out its special traits in clinical implication.

## Methods

A meta-analysis conformed with standards set forth by the Preferred Reporting Items for Systematic Reviews and Meta-Analyses (PRISMA) statement (36).

### Search strategy

PubMed, Embase, and Medline were systematically searched from the establishment of databases up to December 2021. The search strategy was edited following the principle of each database and included keywords related to CRP, AMI, and PCI. Previous meta-analyses and other reviews related to the topic were reviewed to identify studies not included in this search strategy. We also scanned the bibliographies of the included articles and relevant reviews for further reference.

### Inclusion and exclusion criteria

After removing duplicates, titles and abstracts were screened to identify potentially relevant studies. All potentially relevant studies proceeded to full-text review by either reviewer independently and studies that met all the following criteria were included as follows: (1) the study was designed as randomized studies, prospective, or retrospective observational design studies; (2) the study population was patients with AMI (including STEMI and NSTEMI) treated with PCI; (3) the target variant was CRP and concrete information of CRP including cut-off, patient number in different groups, measuring methodology and time was available; and (4) the study reported follow-up duration in detail and outcomes of patients.

Exclusion criteria as follows: (1) articles were reviews, animal studies, laboratory studies, conference documents, and letters; (2) the population of studies included patients who suffered from other coronary diseases like stable or unstable angina; (3) patients were treated with other therapies like conservative medication, thrombolysis, or coronary artery bypass graft; (4) patients were not divided into groups by CRP or cut-off was unavailable; (5) incidence of outcomes in each group could not be directly attained or indirectly calculated; and (6) studies were without access to full text for quality assessment or data extraction.

Any discrepancy was settled by getting through full texture to reach a consensus.

### Data extraction

Two of the authors independently performed data extraction, using a standard data extraction form that contained publication details (name of the first author, year of publication, and region), characteristics of the studied population (sample size, age, and gender distribution), and traits of CRP (high-sensitivity, cut-off, and time of blood taking), the follow-up, and outcomes.

### Outcomes

The end points were as follows: all-cause mortality, cardiovascular death, MACE, heart failure, recurrent myocardial infarction, restenosis, and revascularization. All-cause mortality was defined as the death without specific causes. MACE was a composite definition usually consisting of death, cardiovascular death, recurrent MI, revascularization, stroke, and heart failure. In some studies, more adverse events were composited into MACEs like cardiopulmonary resuscitation and malignant arrhythmia; on the contrary, some selected only few events as MACE. Restenosis is defined as a narrowing of vessel diameter greater than 50% to that of the reference vessel (37), consisting of stent restenosis and in-stent restenosis (38). The former is defined as the presence of an acute coronary syndrome with angiographic or autopsy evidence of thrombus or occlusion. In this meta-analysis, we did not conduct the subgroup analysis between different types of restenosis due to the limitation of research quantity.

### Data analysis

All analyses were conducted using the Review Manager 5.4. The results of our meta-analysis were present as adjusted ORs with 95% confidence intervals (CI). The heterogeneity was presented with estimation using the *I*^2^ statistic. When *I*^2^ statistic is less than 50%, the measured data were pooled in the study and analyzed using a fixed-effects meta-analysis model with inverse variance weighting. On the contrary, a random-effects model is selected. All *p*-values were two-tailed with the statistical significance set at 0.05.

## Results

### Study selection

**FIGURE 1:**
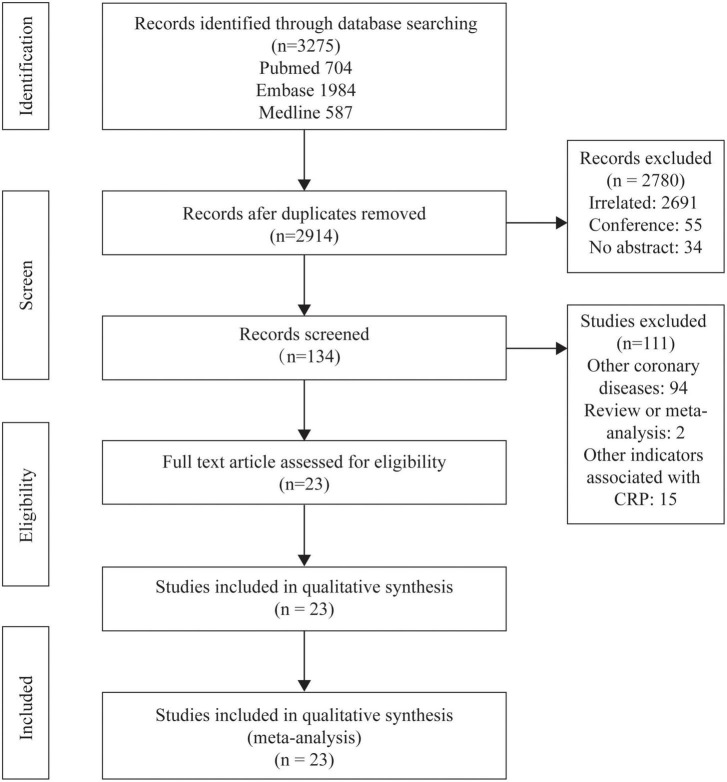
Flowcharts showing relevant studies.

The study selection process is shown in Figure 1. After selection and evaluation, 23 studies were eventually eligible for this meta-analysis (5, 25–34, 39–50) (Table 1). Overall, there were 18,715 patients involved in our analysis, 12,109 in the elevated CRP group and 6,606 in the normal CRP group. The follow-up period varied from in-hospital to 48.3 months. CRP in 17 (5, 27, 28, 30–34, 39, 41, 43–47, 49, 50) studies were measured with high-sensitivity methodology and in one (29) not mentioned. The cut-off values in different experiments were not exactly the same, while 2 mg/L (5, 28, 30, 32, 43), 3 mg/L (25, 27, 33, 40, 41, 45, 49), and 5 mg/L (31, 39, 42) were the most common cut-off values in included experiments. Instead of presupposed cut-off, some studies divided patients by points of bisection (34, 46–48, 50), trisection (28, 30), quadrisection (41, 44) according to CRP value. The diversion point ranged from 2 to 9 mg/L (46) and was selected as the cut-off. The majority of population was originated from Europe (5, 29, 32, 41, 48) and Asian (25–28, 30, 31, 33, 34, 39, 40, 42–47, 49, 50).

**TABLE 1:** Characteristics of studies included in the meta-analysis.

First author	Year	Region	Number of patients included (age, %males)	Types of MI	Follw-up*	hs-CRP	Cut-off (mg/L)	Measuring time	Endpoints	Results
Bicciré, F. G.	2021	Europe	220 (68 ± 12.1, 58%)	STEMI	In-hospital	N/A	17	Peak value	MACEs	Low SA is not infrequent in patients admitted for STEMI treatment and is associated with worse outcomes independently from troponin and CRP levels.
Wang, Y.	2021	Asian	2318 (58.8 ± 11.9, 79.8%)	STEMI	30 months	Yes	2.0	After PCI	MACEs	Systemic inflammation (hsCRP≥2mg/L) can modulate Lp(a)-associated MACE risk in STEMI-PCI patients.
Świątkiewicz, I.	2021	Europe	204 (56.17, 76.5%)	STEMI	6 months	Yes	2.0	1 month after discharge	HF	Persistent elevation in CRP concentration post-STEMI can serve as a risk marker and aid in identifying patients at increased risk of HF and HF-related mortality in multi-year period.
Jiang, H.	2021	Asian	203 (60.35 ± 10.24, 66.0%)	STEMI	12 months	Yes	2.0	Within 72 h inhospitalization	All-cause death, cardiovascular death, HF, reMI	The presence of hs-CRP was not only significantly associated with platelet inhibition function, but was also a prognostic marker in STEMI.
Kang, D. O.	2019	Asian	4410 (62.7 ± 12.4, 77.3%)	STEMI and NSTEMI	36 months	Yes	3.0	On admission	All-cause death, cardiovascular death, MACEs, reMI	The prognostic impact of elevated hs-CRP at baseline was most evident during the first 6 months after AMI.
Her, A. Y.	2017	Asian	146 (57.1 ± 12.4, 81.4%)	STEMI	24 months	Yes	2.7	On admission	MACEs	Elevated hs-CRP and NLR levels were significantly associated with MACE in STEMI patients successfully treated with DES and had incremental predictive values over conventional risk factors.
Shin, H. C.	2017	Asian	381 (61.64 ± 11.0, 76.1%)	STEMI and NSTEMI	24 months	No	7.6	Before PCI	All-cause death, cardiovascular death, MACEs, reMI, restonisis	Elevated levels of both NLR and CRP are associated with increased risk of long-term mortality in AMI patients with PCI.
Wang, C. H.	2015	Asian	241 (63.70 ± 11.96, 77.6%)	STEMI	48.3 months	Yes	3.0	After PCI	All-cause death, cardiovascular death, MACEs, reMI, restonisis	Renal dysfunction and elevated hsCRP predict a high long-term incidence of MACE in patients with acute STEMI with primary PCI, with the combination being of prognostic significance for long-term mortality and MI in these patients.
Shacham, Y.	2015	Asian	562(62 ± 13, 80%)	STEMI	1 month	Yes	9.0	Before PCI	All-cause death and HF	Admission serum hs-CRP level (>9 mg/l) is an independent predictor for AKI following primary PCI in STEMI patients.
Jian-Wei, Z.	2014	Asian	1452(64.50 ± 13.29, 81.68%)	STEMI	In-hospital	Yes	3.0	Before PCI	All-cause death and MACEs	Higher baseline hsCRP level (>6.50 mg/L) was an independent and significant predictor of CIN after p-PCI. A high level of hsCRP was strongly associated with inhospital mortality and composite MACE.
He, Y. T.	2013	Asian	220(62.38 ± 12.28, 82.73%)	STEMI	In-hospital	Yes	6.26	On admission	All-cause death	hs-CRP is positively correlated with CIN incidence.
Kim, K. H.	2013	Asian	5123(62.94 ± 13.38, 74.12%)	STEMI	24 months	Yes	3.0	On admission	MACEs	For STEMI patients with a long ischemic time (≥6 hours), an elevated level of hs-CRP is a poor prognostic factor of long-term cardiovascular outcomes.
Ahmed, K.	2012	Asian	5647(60.4 ± 12.2, 75.2%)	STEMI and NSTEMI	12 months	Yes	2.0	On admission	All-cause death and MACEs	Higher baseline hs-CRP level (≥4.08 mg/dL) in overweight/obese AMI patients showed significant association with 12-month all-cause mortality independent of other prognostic markers.
Schoos, M. M.	2011	Europe	258(60.83, 75.97%)	STEMI	36 months	Yes	2.0	Before pPCI	All-cause death, cardiovascular death, reMI and restenosis	BMS implantation should be preferred when hs-CRP is <2 mg/L and DES when hs-CRP is >2 mg/L to decrease long-term adverse outcomes including stent thrombosis in patients with STEMI treated with pPCI.
Damman, P.	2011	Europe	1034(62 ± 13, 73%)	STEMI	30 months	No	7.0	Before pPCI	All-cause death	The sole use or addition of a multimarker to a model including established risk factors improves the prediction of mortality in STEMI patients undergoing PPCI.
L. I. Gui-Hua	2009	Asian	84(58 ± 11, 65.48%)	STEMI	3–12 months	No	5.0	Within 6h onset	Cardiovascular death, MACEs and HF	The CRP levels within 6h after attack of AMI can be taken as one of the indexes to predict the prognosis of PCI.
Ortolani, P.	2008	Europe	758(68.00, 70.45%)	STEMI	in-hospital	Yes	3.1	On admission	All-cause death, MACEs, reMI and restenosis	hs-CRP levels at admission independently predict in-hospital and long-term clinical outcome, potentially negatively influencing survival.
Jeong, Y. H.	2008	Asian	207(57.3 ± 12.0, 81.6%)	STEMI	12 months	Yes	5.0	On admission	All-cause death, MACEs, HF and reMI	cTnI and hs-CRP levels on admission give no addictive information on classic TIMI risk score for predicting long-term cardiovascular outcomes in STEMI patients treated with primary DES implantation and intensive medical therapy.
Yip, H. K.	2005	Asian	146(60.0 ± 10.8, 86.3%)	STEMI	1 months	Yes	2.37	Before PCI	All-cause death, MACEs and restenosis	Prospective evaluation ofthe hsCRP in STEMI of onset < 6 h allows accurate risk stratification of individuals at risk of 30-day MACE after primary PCI.
Liu, Jun	2004	Asian	76(N/A)	STEMI	6 months	No	3.0	Within 6 h onset	All-cause death, MACEs and reMI	CRP levels within six hours after the onset of AMI might predict early and late outcome after primary PCI.
Magadle, R.	2004	Asian	230(63,58 ± 8.80, 75.22%)	STEMI	12 months	Yes	5.0	Before PCI	All-cause death, MACEs, reMI and restenosis	Preprocedural serum CRP level might be considered a powerful predictor of early but not late complications in patients undergoing PTCA/stent procedures.
Hong, Y. J.	2003	Asian	208(59.43 ± 9.96, 79.81%)	STEMI	12 months	No	1.0	On admission	All-cause death, MACEs, reMI and restenosis	An leevated CRP is an independent prognostic marker in patients with acute myocaridal infarction after primary or rescue PCI.
Tomoda, H.	2000	Asian	234(62.42 ± 10, 77.35%)	STEMI	6 months	No	3.0	Within 6 h onset	All-cause death, MACEs, reMI and restenosis	CRP levels within 6 h after the onset of AMI reflect the vulnerability of culprit coronary lesions and predict adverse coronary events after primary PTCA/stenting.

*The longest follow time of studies.

### C-reactive protein and outcomes

**FIGURE 2:**
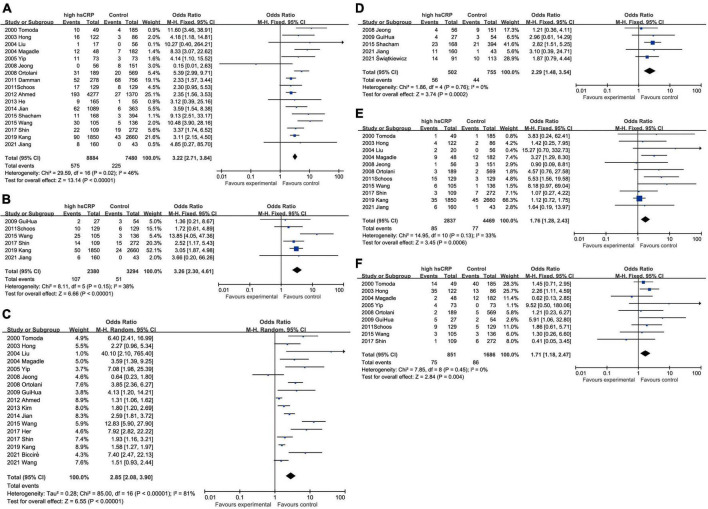
Forest plots of studies assessing the association between elevated CRP and different outcomes among patients with AMI with PCI: **(A)** CRP and all-cause mortality; **(B)** CRP and cardiovascular mortality; **(C)** CRP and MACEs; **(D)** CRP and heart failure; **(E)** CRP and recurrent myocardial infarction; and **(F)** CRP and restenosis.

Elevated CRP was associated with increased all-cause mortality (OR: 3.22, 95% CI [2.71, 3.84], *p* < 0.00001, *I*^2^ = 46%) (25–28, 31–34, 39–41, 43, 44, 46–49) and cardiovascular mortality (OR: 3.26, 95% CI [2.30, 4.61], *p* < 0.00001, *I*^2^ = 34%) (27, 28, 32–34, 42). In addition to mortality, elevated CRP were also related to higher incidence of various malignant cardiovascular events, such as MACEs (OR: 2.85, 95% CI [2.08, 3.90], *p* < 0.00001, *I*^2^ = 81%) (25–27, 29–31, 33, 34, 39–43, 45, 47, 49, 50), heart failure (OR: 2.29, 95% CI [1.48, 3.54], *p* = 0.0002, *I*^2^ = 0%) (5, 28, 31, 42, 46), recurrent myocardial infarction (OR: 1.76, 95% CI [1.28, 2.43], *p* < 0.001, *I*^2^ = 33%) (25–28, 31–34, 39–41), and restenosis (OR: 1.71, 95% CI [1.18, 2.47], *p* = 0.004, *I*^2^ = 0%) (25, 26, 32–34, 39, 41, 42, 47). All these results have shown the potential of CRP as the prognostic biomarker for multiple outcomes in patients with AMI after PCI (Figure 2). With respect to the situation that different variants like duration of follow-up, traits of CRP, and racial distributions may lead to different outcomes, we processed the data in the trials and conducted subgroup analyses to shrink heterogeneity, while sensitivity analysis verified the stability of results (Supplementary Table 1).

### Subgroup analysis

#### C-reactive protein and all-cause mortality

**FIGURE 3:**
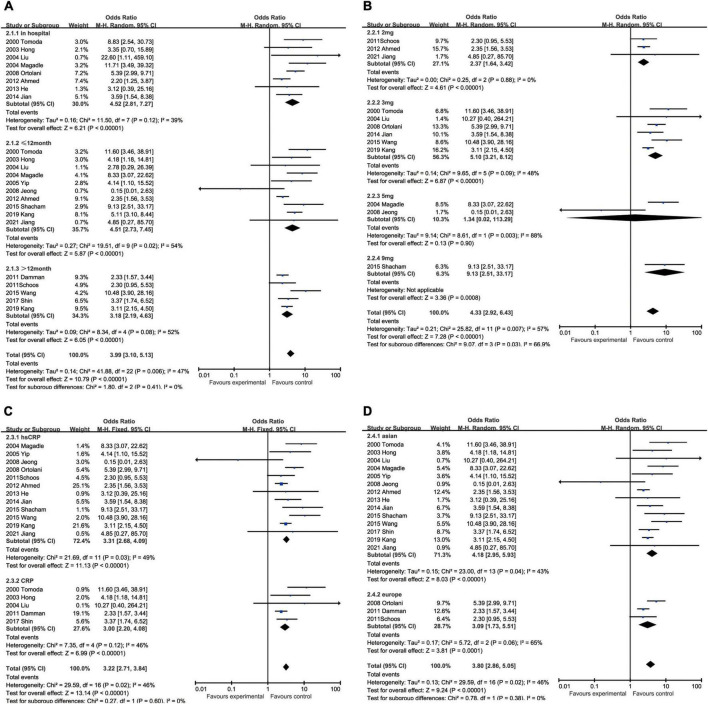
Forrest plots of subgroup analysis assessing all-cause mortality associated with increased CRP: **(A)** subgroups based on follow-up; **(B)** subgroups based on cut-off value of hsCRP; **(C)** subgroups based on CRP and hsCRP; and **(D)** subgroups based on ethnicities.

All-cause mortality was one of the most concerning outcomes of AMI. Increased CRP was invariably a powerful predictor to all-cause mortality in hospital (OR: 4.52, 95% CI [2.81, 7.27], *p* < 0.00001, *I*^2^ = 39%), <12 months (OR: 4.51, 95% CI [2.73, 7.45], *p* < 0.00001, *I*^2^ = 54%) and ≥12 months (OR: 3.18, 95% CI [2.19, 4.63], *p* < 0.00001, *I*^2^ = 52%) (Figure 3A). When we took the marginal value of CRP into subgroup analysis, 3 mg/L (OR: 5.10, 95% CI [3.21, 8.12], *p* < 0.00001, *I*^2^ = 48%) was the most frequently chosen cut-off value and had been shown a more powerful capacity than 2 mg/L (OR: 2.37, 95% CI [1.64, 3.42], *p* < 0.00001, *I*^2^ = 0) and 5 mg/L (OR: 1.34, 95% CI [0.02, 113.29], *p* = 0.90, *I*^2^ = 88%) to predict the mortality. Five milligrams per liter exhibited no statistical significance. Although 9 mg/L had shown to be the best predictor (OR: 9.13, 95% CI [2.51, 33.17], *p* = 0.008), only one study was included. Besides, elevated hsCRP (OR: 3.31, 95% CI [2.68, 4.09], *p* < 0.00001, *I*^2^ = 49%) had slight advantage than CRP (OR: 3.00, 95% CI [2.20, 4.08], *p* < 0.00001, *I*^2^ = 46%). In addition, the upregulated CRP was shown to be less associated with mortality in European (OR: 3.09, 95% CI [1.73, 5.51], *p* < 0.00001, *I*^2^ = 43%) than in Asian (OR: 4.18, 95% CI [2.95, 5.93], *p* < 0.00001, *I*^2^ = 65%) (Figure 3).

#### C-reactive protein and cardiovascular mortality

**FIGURE 4:**
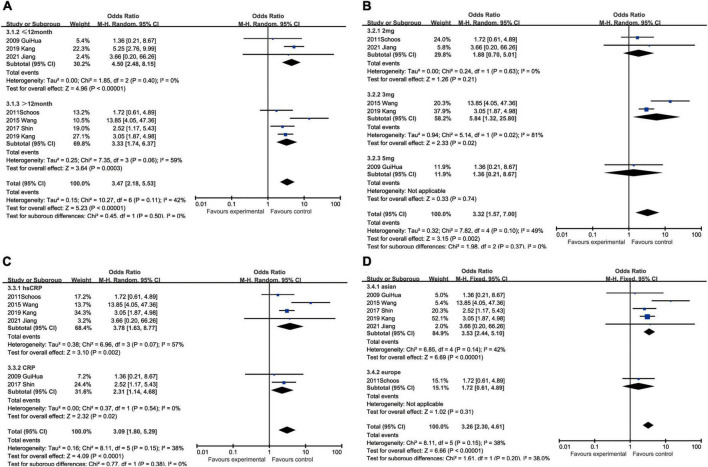
Forrest plots of subgroup analysis assessing cardiovascular mortality associated with increased CRP: **(A)** subgroups based on follow-up; **(B)** subgroups based on cut-off value of hsCRP; **(C)** subgroups based on CRP and hsCRP; and **(D)** subgroups based on ethnicities.

The augment of CRP amplified the possibility of cardiovascular death within 12 months after hospitalization (OR: 4.50, 95% CI [2.48, 8.15], *p* < 0.00001, *I*^2^ = 0) and the influence still existed when the follow-up was prolonged to more than 12 months (OR: 3.33, 95% CI [1.74, 6.37], *p* < 0.00001, *I*^2^ = 59%). With respect to the critical value of CRP, 3 mg/L (OR: 5.84, 95% CI [1.32, 25.80], *p* = 0.02, *I*^2^ = 81%) was usually the most recommended, while the others like 2 mg/L (OR: 1.88, 95% CI [0.70, 5.01], *p* = 0.21, *I*^2^ = 0) and 5 mg/L (OR: 1.36, 95% CI [0.21, 8.67], *p* = 0.74) had no statistical significance. The measuring methodologies did not exert obvious impact on the incidence of cardiovascular death, although hsCRP (OR: 3.78, 95% CI [1.63, 8.77], *p* = 0.002, *I*^2^ = 57%) was presumed to perform better than CRP (OR: 2.31, 95% CI [1.14, 4.68], *p* = 0.02, *I*^2^ = 0). The ethnicity had also an effect on cardiovascular death, while Asian (OR 3.53, 95% CI [2.44, 5.10], *p* < 0.00001, *I*^2^ = 42%) were at higher risk than European (OR: 1.72 [0.61, 4.89], *p* = 0.20) and the latter had no statistical significance (Figure 4).

#### C-reactive protein and major adverse cardiovascular events

**FIGURE 5:**
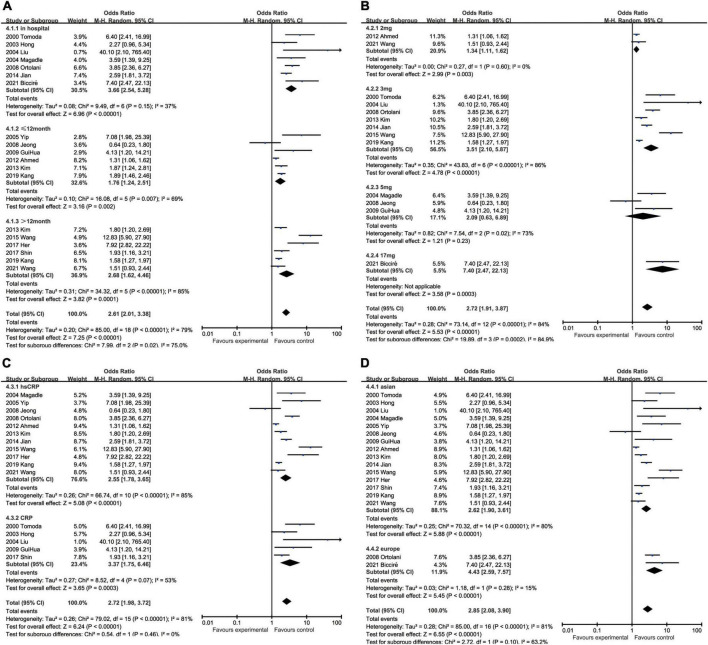
Forrest plots of subgroup analysis assessing MACEs associated with increased CRP: **(A)** subgroups based on follow-up; **(B)** subgroups based on cut-off value of hsCRP; **(C)** subgroups based on CRP and hsCRP; and **(D)** subgroups based on ethnicities.

In regard to MACEs, elevated CRP might result in increased hospitalized malignant events (OR: 3.66, 95% CI [2.54, 5.28], *p* < 0.00001, *I*^2^ = 37%) with medium heterogeneity. We also analyzed the association between ascending CRP and incidence of MACEs within 12 months (OR: 1.76, 95% CI [1.24, 2.51], *p* = 0.002, *I*^2^ = 69%) or over 12 months (OR: 2.68, 95% CI [1.62, 4.46], *p* = 0.0001, *I*^2^ = 84%), but greater heterogeneity was accompanied with the elongation of follow-up. Three milligrams per liter (OR: 3.51, 95% CI [2.10, 5.87], *p* < 0.00001, *I*^2^ = 86%) was a superior threshold in dividing patients with AMI with higher or lower risk of MACEs, while mediocre performances exhibited in other cut-off such as 2 mg/L (OR: 1.34, 95% CI [1.11, 1.63], *p* = 0.003, *I*^2^ = 0) and 5 mg/L (OR: 2.09, 95% CI [0.63, 6.89], *p* = 0.23, *I*^2^ = 73%) which had no statistical significance. As for CRP measuring methodology, CRP (OR: 3.37, 95% CI [1.75, 6.46], *p* = 0.0003, *I*^2^ = 53%) was more efficient to predict MACEs than hsCRP (OR: 2.55, 95% CI [1.78, 3.65], *p* < 0.00001, *I*^2^ = 85%). Considering ethnics, the subgroup analysis implied that Caucasian patients (OR: 4.43, 95% CI [2.59, 7.57], *p* < 0.00001, *I*^2^ = 15%) were more susceptible to MACEs than Asian (OR: 2.62, 95% CI [1.90, 3.61], *p* < 0.00001, *I*^2^ = 80%) (Figure 5).

#### C-reactive protein and heart failure

**FIGURE 6:**
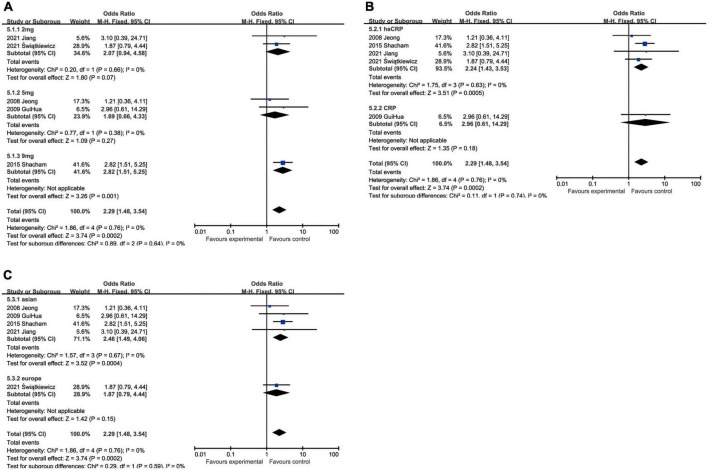
Forrest plots of subgroup analysis assessing heart failure associated with increased CRP: **(A)** subgroups based on cut-off value of hsCRP; **(B)** subgroups based on CRP and hsCRP; and **(C)** subgroups based on ethnicities.

Although heart failure was reported in only five studies containing 1,257 patients, the marginal values of hsCRP were variably selected. When we put different cut-off values into different subgroups for analysis, no statistical significance could be found in some subgroups, namely 2 mg/L group (OR: 2.07, 95% CI [0.94, 4.58], *p* = 0.07, *I*^2^ = 0) and 5 mg/L group (OR: 1.69, 95% CI [0.66, 4.33], *p* = 0.27, *I*^2^ = 0). As the critical value was upregulated to 9 mg/L (OR: 2.82, 95% CI [1.51, 5.25], *p* = 0.001), statistical significance was eventually exhibited. In addition, hsCRP (OR: 2.24, 95% CI [1.43, 3.53], *p* = 0.0005, *I*^2^ = 0) could be a predictor of incidence of heart failure, while CRP (OR: 2.96, 95% CI [0.61, 14.29], *p* = 0.18) had no statistical significance. Asian patients (OR: 2.46, 95% CI [1.49, 4.06], *p* = 0.0004, *I*^2^ = 0) were under statistically significant risk to heart failure, comparing with European (OR: 1.87, 95% CI [0.79, 4.44], *p* = 0.15) whose subgroup analysis result had no statistical significance (Figure 6).

#### C-reactive protein and recurrent myocardial infarction

**FIGURE 7:**
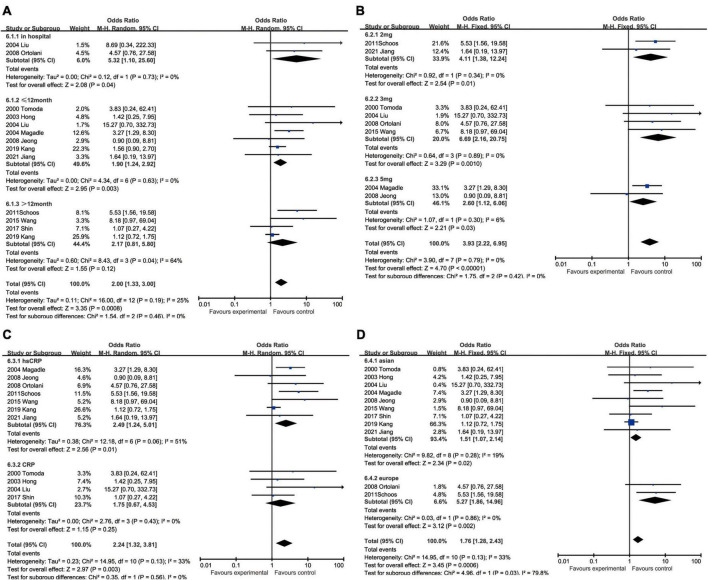
Forrest plots of subgroup analysis assessing recurrent myocardial infarction associated with increased CRP: **(A)** subgroups based on cut-off value of hsCRP; **(B)** subgroups based on CRP and hsCRP; **(C)** subgroups based on ethnicities; and **(D)** subgroups based on ethnicities.

With regard to recurrent myocardial infarction, subgroup analysis indicated that enhanced CRP could foresee soaring incidence of recurrent myocardial infarction in hospitalization (OR: 5.32, 95% CI [1.10, 25.60], *p* = 0.04, *I*^2^ = 0), within 12 months (OR: 1.90, 95% CI [1.24, 2.92], *p* = 0.003, *I*^2^ = 0), while CRP showed no statistical significance over 12 months (OR: 2.17, 95% CI [0.81, 5.80], *p* = 0.12, *I*^2^ = 64%). Three milligrams per liter (OR: 6.69, 95% CI [2.16, 20.75], *p* = 0.0010, *I*^2^ = 0) had a satisfactory performance as a cut-off value of CRP, whereas 2 mg/L (OR: 4.11, 95% CI [1.38, 12.24], *p* = 0.01, *I*^2^ = 0) and 5 mg/L (OR: 2.60, 95% CI [1.12, 6.06], *p* = 0.03, *I*^2^ = 6%) were also equal to predicting recurrent myocardial infarction. Elevated hsCRP (OR: 2.49, 95% CI [1.24, 5.01], *p* = 0.01, *I*^2^ = 51%) was considered to be a potential risk factor, especially comparing to CRP (OR: 1.75, 95% CI [0.67, 4.53], *p* = 0.25, *I*^2^ = 0) measured by normal sensitivity methodology which had no statistical significance. When we focused on the impact of ethnics on heterogeneity, European (OR: 5.27, 95% CI [1.86, 14.96], *p* = 0.002, *I*^2^ = 0) were more susceptible to recurrent myocardial infarction than Asian (OR: 1.51, 95% CI [1.07, 2.14], *p* = 0.02, *I*^2^ = 19%) (Figure 7).

#### C-reactive protein and restenosis

**FIGURE 8:**
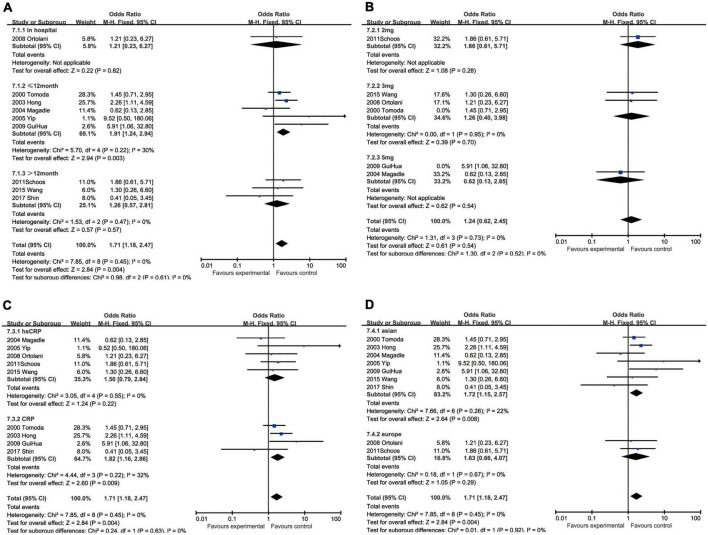
Forrest plots of subgroup analysis assessing restenosis associated with increased CRP: **(A)** subgroups based on cut-off value of hsCRP; **(B)** subgroups based on CRP and hsCRP; **(C)** subgroups based on ethnicities; and **(D)** subgroups based on ethnicities.

The subgroup analysis demonstrated that the predicting capacity of increased CRP had a relatively narrow time window within 12 months (OR: 1.91, 95% CI [1.24, 2.94], *p* = 0.003, *I*^2^ = 30%), whereas neither in hospitalization (OR: 1.21, 95% CI [0.23, 6.27], *p* = 0.82) nor over 12 months (OR: 1.26, 95% CI [0.57, 2.81], *p* = 0.57, *I*^2^ = 0) had statistical significance. None of the cut-off performed statistically significant, namely 2 mg/L group (OR: 1.86, 95% CI [0.61, 5.71], *p* = 0.28), 3 mg/L (OR: 1.39, 95% CI [0.76, 2.55], *p* = 0.28, *I*^2^ = 0), 5 mg/L (OR: 1.59, 95% CI [0.60, 4.22], *p* = 0.35, *I*^2^ = 73%) and 7.6 mg/L (OR: 0.41, 95% CI [0.05, 3.45], *p* = 0.54). The subgroup analysis could help us make the decision between CRP (OR: 1.82, 95% CI [1.16, 2.86], *p* = 0.009, *I*^2^ = 32%) and hsCRP (OR: 1.50, 95% CI [0.79, 2.84], *p* = 0.22, *I*^2^ = 0) as a prognostic factor for restenosis for reason of that the latter had no statistical significance. Ethnically, CRP could predict restenosis in Asians ((OR = 1.72, 95% CI [1.15, 2.57], *p* = 0.008, *I*^2^ = 22%), but not in Europeans (OR: 1.63, 95% CI [0.66, 4.07], *p* = 0.29, *I*^2^ = 0) (Figure 8).

## Discussion

Briefly summarizing the results of our meta-analysis, the elevation of CRP is significantly associated with the boost in multiple adverse prognoses for the patients with AMI undergoing PCI, especially in all-cause mortality and cardiovascular death. Furthermore, it is noteworthy that increased CRP has more prognostic value in hospitalization and short-term outcomes and the Asian group.

Recent years have emerged plenty of studies investigating diverse post-infarction prognostic inflammatory biomarkers originating from either inflammation infiltration or plasma inflammatory factors. Biomarkers based on inflammatory infiltration have been verified from simple white blood cell counts (51) to counts (8) or ratios (52, 53) of a different specific subclass of innate immune cells. NLR was most reported to be related to the hospital and long-term prognosis of patients with STEMI after PCI (12). Scoring systems have also been developed from white blood cell counts, such as SII (54) foreseeing no-reflow phenomenon in patients with STEMI after PCI (13). Nevertheless, these biomarkers cannot get rid of the disadvantages of WBC counts that are easily influenced by race and sex and it is difficult to establish a reference range for the whole population (52) and are in need of further verification. Though single-cell proteomic and transcriptomic analyses uncovered distinct features of novel subclasses in atherosclerotic lesions (55), it is promising but remains to be explored in clinical transformation looking forward to high-quality randomized controlled trials.

Comparing to inflammatory infiltration, conventional plasma inflammatory mediators are routinely measured in clinical practice and have been verified as prospective prognostic biomarkers. For example, IL-1b was associated with 90-day all-cause mortality, cardiovascular mortality, and MACEs but defected in predicting nonfatal cardiovascular events (15), while Kilic et al. (16) suggested cytokine concentrations and proinflammatory to anti-inflammatory cytokine ratios as markers of a high risk of new coronary events. Moreover, comprehensive studies or reviews (16, 19, 56–58) were conducted comparing the prognostic performance of multiple conventional inflammatory factors like IL-6, IL-10, TNF-α, and CRP. Among these mediators, CRP has exhibited a predominant power. Lippi et al. (56) compared prognostic significance of 12 cytokines or growth factors in patients with myocardial ischemia and among these biomarkers, CRP displayed the most notable risk estimates identifying potential post-infarction heart failure victims. Ritschel et al. (57) found that neither IL-6 nor sgp130 levels were related to future events by cox regression models, but patients with elevated CRP levels had a higher risk of death. Furthermore, compared with part of inflammatory factors, CRP has its own advantages such as no diurnal variation, insensitivity to external factors like diet and stable measured value (17). In the last decades, elevated CRP has been studied and verified in plenty of cohorts and is widely acknowledged as a cardiovascular risk factor. In the previous meta-analysis by Mincu et al. (35), high preprocedural CRP levels prognosed a significant increase of the incidence of in-hospital and follow-up adverse end points. However, recent years novel cohorts have been reported and associated CRP with more post-infarction events, such as heart failure (5).

In the current study analysis, the subgroup provided us new prospective on the clinical application of the relationship between elevated CRP and poor AMI prognosis. First, though CRP displayed a favorable predicting capacity of hospitalization, short-term (<12 months) or long-term (>12 months) prognosis, it differed between end points. Comparing with all outcomes, CRP can better foresee hospitalization incidence of MACEs and recurrent myocardial infarction and short-term restenosis while both all-term all-cause mortality and cardiovascular mortality. Świątkiewicz et al. (5) had reported that persistent elevation in CRP was associated with increased risk of hospitalization for HF and HF-related mortality in long-term (>6 years) follow-up, which further completed the function of CRP as a predictor. Second, as the most adopted thresholds, 2 and 3 mg/L can both serve as an optimal cut-off value for post-infarction adverse prognosis. In general, standard clinical assays for CRP with the normal sensitivity range from 3 to 8 mg/L (59), which limits its effective application to vascular risk prediction. To solve the limitation of CRP, high-sensitive CRP with excellent fidelity and reproducibility was developed and its comparability was proven (60). In the current study, hsCRP has better performance in predicting all-cause mortality, cardiovascular mortality, and recurrent myocardial infarction. However, CRP had its superiority in MACEs, heart failure, and restenosis, which implies unknown underlying impact factors in need of further investigation. Third, elevated CRP is a more important sign of adverse prognosis in Asian patients than in European. With respect to ethnic groups, we found that CRP had better performance in predicting the all-cause mortality, cardiovascular mortality, the incidence of heart failure and restenosis in Asians, and the incidence of MACEs and recurrent myocardial infarction in Europeans, while it was not statistically significant to predict the cardiovascular mortality, the incidence of heart failure and restenosis in Europeans. The diversity might originate from the differences in genetic backgrounds and environments, different including criteria, and selection biases (61). A meta-analysis of 7,703 subjects has shown CRP gene polymorphisms associated with decreased risk of MI among Asian populations, while no statistical significance was found among Caucasian populations (62).

There are also limitations in our study. First, as is known, there are two types of AMI (i.e., STEMI and NSTEMI). The majority of our including studies focused on the relationship between CRP and STEMI. Although three studies (27, 34, 43) incorporate patients with NSTEMI, the detailed information could not be extracted from the source articles. In consideration of different inflammation conditions (58) and outcome profiles (63) under STEMI or NSTEMI, these diversities may lead our results to be less appropriate for NSTEMI. Second, in-depth studies have been conducted in an attempt to improve the sensitivity and specificity of CRP, attempting to adjust or combine hsCRP with its change velocity (64, 65), cTnT (66), albumin (67–69), and fibrinogen (70). These studies extended the practicability of CRP but are not included in our study due to lack of enough high-quality cohorts. Finally, there is publication bias existing in all-cause mortality and MACEs. As these studies were carefully selected following PRISMA and the results remain stable after sensitivity analysis, we preferred not to delete studies.

In brief, the current meta-analysis suggests that CRP is a prospective predictor of the diverse adverse prognoses in patients with AMI undergoing PCI. CRP had better performance in predicting plenty of hospitalization and short-term (<12 months) adverse prognoses than long-term prognoses and it is noteworthy that elevated CRP in Asian patients had more predictive value than in European.

## Data availability statement

The original contributions presented in this study are included in the article/Supplementary material, further inquiries can be directed to the corresponding author.

## Author contributions

HZ conceptually designed the work. LD and YJ critically reviewed the manuscript. MD provided constructive recommendations in data processing and article polishing. SL and HJ conducted the main work and prepared the manuscript. All authors contributed to the article and approved the submitted version.
