# Supplementary figures and images for: Evaluation of C-reactive protein as predictor of adverse prognosis in acute myocardial infarction after percutaneous coronary intervention: A systematic review and meta-analysis from 18,715 individuals
Source: Front Cardiovasc Med. 2022 Nov 16;9:1013501. doi: 10.3389/fcvm.2022.1013501 (PMC9708737; doi:10.3389/fcvm.2022.1013501)

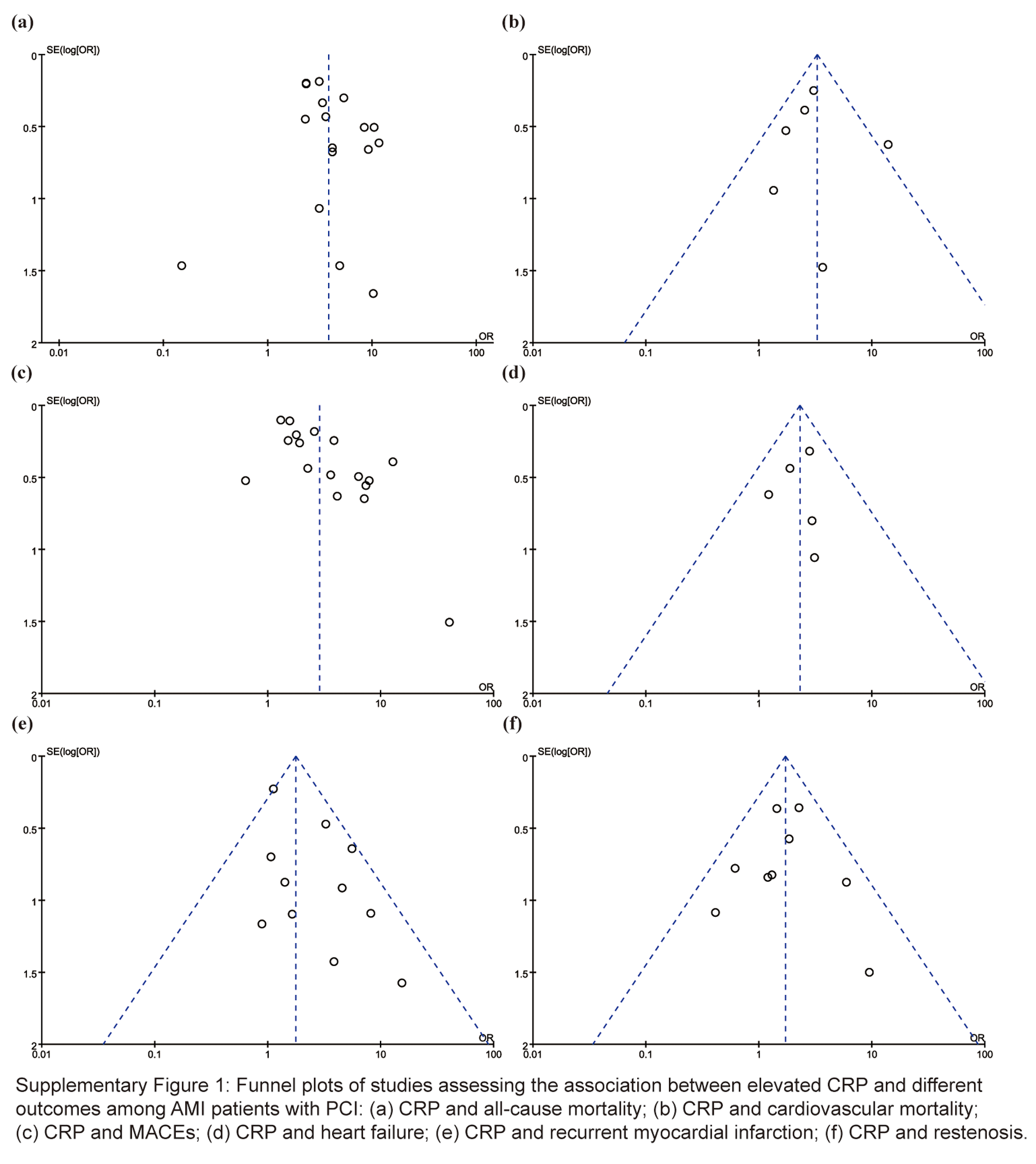

Supplement: Supplementary file 1 [file Image_1.TIF]
